# Climate‐Associated Genetic Variation and Projected Genetic Offsets for *Cryptomeria japonica* D. Don Under Future Climate Scenarios

**DOI:** 10.1111/eva.70077

**Published:** 2025-02-06

**Authors:** Kentaro Uchiyama, Tokuko Ujino‐Ihara, Katsuhiro Nakao, Jumpei Toriyama, Shoji Hashimoto, Yoshihiko Tsumura

**Affiliations:** ^1^ Department of Forest Molecular Genetics and Biotechnology Forestry and Forest Products Research Institute Tsukuba Ibaraki Japan; ^2^ Kansai Research Center Forestry and Forest Products Research Institute Kyoto Kyoto Japan; ^3^ Kyushu Research Center Forestry and Forest Products Research Institute Kumamoto‐city Kumamoto Japan; ^4^ Department of Forest Soils Forestry and Forest Products Research Institute Tsukuba Ibaraki Japan; ^5^ Graduate School of Agricultural and Life Sciences The University of Tokyo Bunkyo‐ku Tokyo Japan; ^6^ Faculty of Life and Environmental Sciences University of Tsukuba Tsukuba Ibaraki Japan

**Keywords:** climate change, *Cryptomeria japonica* D. Don, genetic offset, gradient Forest, landscape genomics, local adaptation

## Abstract

Revealing the spatial distribution of adaptive genetic variation is both a challenging and crucial task in evolutionary ecology, essential for understanding local adaptation within species, and in management, for predicting species responses to future climate change. This understanding is particularly important for long‐lived tree species, which may not be able to migrate quickly enough to adapt to rapid climate changes and may need to rely on their standing genetic variation. In this study, we focused on *Cryptomeria japonica* , a major component of Japan's temperate forests and an important forestry species adapted to the humid environment of monsoon Asia. We extracted climate‐associated genetic variation from the entire genome and evaluated its distribution and vulnerability under future climate scenarios using spatial modeling techniques. We analyzed 31,676 high‐quality SNPs from 249 individuals across 22 natural populations of *C. japonica* , covering its entire distribution range. We identified 239 candidate climate‐associated SNPs and found winter temperature, summer precipitation, and winter precipitation as the most significant factors explaining the genetic variation in these SNPs. The climate‐associated genetic variation deviated from non‐associated (neutral) genetic variation in the opposite (the Sea of Japan and Pacific Ocean) sides of Japanese archipelago, suggesting natural selection of different climate conditions in these regions. Difference in estimated allele frequency at the climate‐associated loci (genetic offset) between the present and future (2090 in the SSP5‐8.5 scenario) climate conditions was predicted to be larger in three areas (not only southwestern Japan but also coastal area on the Sea of Japan side and inland area on the Pacific Ocean side in northeastern Japan). This prediction implies the discrepancy between standing genetic variation at the present and that adaptive to the future climate in these areas, which underscores the necessity for proactive management to adjust the adaptive genetic variation.

## 1 Introduction

Climate change poses significant challenges to forest ecosystems, particularly for long‐lived tree species like *Cryptomeria japonica* . These species are keystone components of their ecosystems, providing stability and vital ecosystem services. However, rapidly shifting climatic conditions increase the risk of maladaptation, threatening the productivity, resilience, and ecological roles of such species (Kremer et al. 2012). For forestry management, ensuring the long‐term adaptability of tree species is critical to sustaining both ecosystem stability and forestry practices under future climate scenarios. Addressing these challenges requires a detailed understanding of adaptive genetic variation, its environmental drivers, and the potential impacts of climate change (Chung et al. 2023). Tree species distributed across highly heterogeneous environments often exhibit local adaptation in tree species (Rehfeldt et al. 2003, 1999; Risk et al. 2021). This suggests that adaptive genetic variation associated with environmental responses may underlie the low genetic differentiation typically observed between tree populations (often less than a few percent) using neutral markers (Hamrick, Godt, and Sherman‐Broyles 1992). However, for tree species, the climatic factors acting as selective pressures and the spatial distribution of adaptive genetic variation can vary significantly by species and landscape, making generalization challenging (Feng and Du 2022).

Recent advancements in high‐resolution climate data, global climate models (Flato et al. 2014), and landscape genomics techniques have enabled researchers to detect adaptive genetic variations through genome‐environment association analyses (Caye et al. 2019; Gautier 2015; Günther and Coop 2013) and map their spatial distribution (Fitzpatrick and Keller 2015). These approaches allow predictions of how adaptive genetic variation will be disrupted under climate change, informing conservation and management strategies.

A key advancement is the concept of genetic offset, which quantifies the gap between current adaptive genetic variation and the variation required under future climates. Methods such as Gradient Forest (Fitzpatrick and Keller 2015), risk of non‐adaptedness (ROMA) (Rellstab et al. 2016), and genomic vulnerability (Bay et al. 2018) estimate this offset, identifying areas where fitness declines are likely under climate change. Such models are especially valuable for long‐lived and low‐mobility species, which are particularly vulnerable to rapid warming and face an increased risk of local extinction. Importantly, spatial modeling of adaptive variation can account for regional adaptation differences, addressing variability in resilience across populations.

*Cryptomeria japonica* is a conifer species belonging to the *Cupressaceae* family. It is naturally distributed in Japan and parts of China, where it is adapted to the humid monsoon climate (Takahara et al. 2023). In Japan, it grows across a wide range of environments, from latitudes spanning 10°, elevations ranging from 0 to 2000 m, and annual precipitation levels between 1000 and 5000 mm (Tsumura 2011), rendering it an ideal model for studying adaptive genetic variation. It is a keystone species in the primary vegetation of Japan's temperate forests, providing ecosystem stability. This species has been utilized in forestry since the Heian period (A.D. 794 to 1185) due to its good growth, ease of processing, and broad environmental adaptability, and has been the most widely utilized tree species historically. Currently, 44% of Japan's artificial forests are occupied by *C. japonica* , accounting for 18% of the forest area (Forestry Agency 2023). The stability of this species is a critical issue from both the perspective of forest ecosystems and forestry use.

**FIGURE 1:**
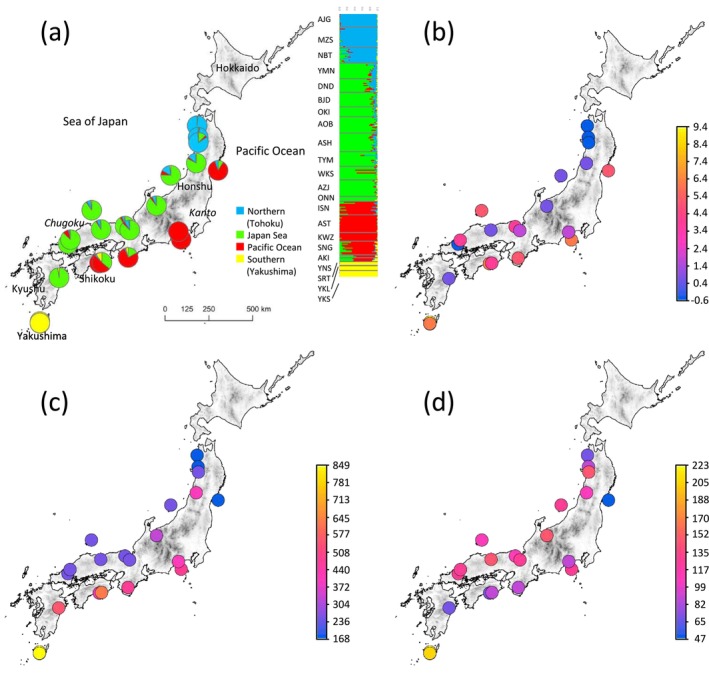
(a) Frequency distribution of four genetic clusters in populations and individuals of *Cryptomeria japonica* identified by popCluster analysis from 16,113 SNPs post‐LD pruning. (b–d) Spatial variation in selected bioclimatic variables: (b) BIO11 (Mean Temperature of Coldest Quarter), (c) BIO13 (Precipitation of Wettest Month), and (d) BIO14 (Precipitation of Driest Month).

Genetic analysis of this species has been conducted for a long time (Tomaru, Tsumura, and Ohba 1992; Tomaru, Tsumura, and Ooba 1994; Tsumura and Tomaru 1999), making it the most advanced conifer species in genome analysis in Japan (Moriguchi et al. 2016, 2012; Tani et al. 2003), with genome information available at the chromosome level (Fujino et al. 2024). Four genetic lineages are known in this species (Tsumura et al. 2014; Uchiyama et al. 2014), including groups adapted to cold environments in northeastern Japan, groups adapted to hot and humid environments at the southern limit, and one group each in regions with significantly different winter climates in eastern and western Japan (Figure 1). Past distribution changes show expansion from the refugium around Tateyama on the Sea of Japan side during the post‐glacial period (Moriguchi et al. 2019), and expansion from northern micro‐refugia is also evident (Kimura et al. 2014). Understanding how this history has contributed to the genetic adaptation of the species is intriguing. Efforts to detect adaptive genetic variation in *C. japonica* were initiated more than a decade ago, primarily focusing on identifying loci associated with environmental gradients and genetic differentiation across populations (Tsumura et al. 2007, 2012, 2014). These studies highlighted evidence of local adaptation, particularly in relation to temperature and precipitation. However, the specific environmental factors shaping the spatial distribution of adaptive genetic variation and the degree of genomic vulnerability to future climatic changes remain largely unknown. Given the detailed research on past transitions through the analysis of neutral loci and the wide range of environments and active gene flow in this species, *C. japonica* is an ideal model for studying adaptive genetic variation along environmental gradients.

This study aims to elucidate the adaptive genetic variation in natural populations of *C. japonica* by analyzing genetic variation across 22 populations, comprising 249 individuals collected from natural forests in Japan. To achieve this, we evaluated the differentiation of adaptive genetic variation among lineages and applied ecological genomics approaches to reveal how adaptive variation is distributed across actual landscapes. Using machine learning techniques, we identified key climatic factors influencing the spatial distribution of adaptive genetic variation. Additionally, we integrated future climate scenarios with Gradient Forest (GF) modeling to predict the spatial distribution of genetic offset, representing the degree of maladaptation expected under climate change.

Specifically, we sought to answer the following questions:

1. Do the four genetic lineages with different histories and distinct habitats also form boundaries in terms of adaptive genetic variation?
2. What are the important climatic factors influencing the distribution of adaptive genetic variation, particularly regarding adaptation to the heavy snowfall environment on the Sea of Japan side?
3. Physiological and ecological studies predict growth decline in low‐precipitation areas of southwestern Japan for *C. japonica* . Does the genetic offset also increase in these regions from the perspective of adaptive genetic variation?

Japan's unique geographical and climatic features make it an exceptional natural laboratory for studying adaptive genetic variation in plants. The country's maritime climate, characterized by high annual precipitation, combined with north–south mountain ranges exceeding 3000 m in elevation, creates a diverse array of environmental gradients. These conditions have facilitated the evolution and persistence of over 5370 vascular plant species (Iwatsuki, Boufford, and Ōba 1993), an unusually high level of biodiversity for temperate regions in the Northern Hemisphere (Mittermeier 2005). Despite this rich biodiversity, the adaptive genetic responses to these environmental gradients are not well understood. This study provides the first comprehensive investigation into the spatial distribution of adaptive genetic variation and its vulnerability to climate change for tree species in the Japanese archipelago, focusing on *C. japonica* as a model.

## 2 Materials and Methods

### 2.1 Sampling and Genomic Data Processing

To comprehensively investigate the genetic diversity and structure of *C. japonica* (Japanese cedar), we analyzed a total of 249 individuals from 22 natural populations across the species' range in Honshu and southern regions of Japan (Figure 1). Fourteen of these populations were previously studied by Tsumura et al. (2014). The climatic values of each population over the past 30 years are shown in Figure 1b–d. These populations span a range of environmental conditions, with winter mean temperatures ranging from −0.6°C to 9.4°C, precipitation in the wettest month from 168 mm to 849 mm, and precipitation in the driest month from 47 mm to 223 mm. DNA was extracted from conifer needle samples using a modified cetyltrimethylammonium bromide (CTAB) method. The quality and concentration of extracted DNA were verified by gel electrophoresis and fluorescence quantification (Quant IT dsDNA HS Assay). For each individual, 500 ng of DNA was prepared for ddRAD‐seq library construction (Peterson et al. 2012). Restriction enzyme digestion was performed using PstI‐HF and SphI‐HF (New England Bio Labs.), followed by ligation of Y‐shaped adaptors using T4 DNA Ligase (Thermo Fisher Scientific). Individual‐specific barcodes (Fluidigm, Sunnyvale, CA) were incorporated by PCR. Following PCR, DNA concentrations were normalized across samples, pooled, and size selected using Blue Pippin gel electrophoresis (Sage Science), targeting an insert size of 350 bp excluding adaptor sequences, with a total library size of 450 bp. The prepared libraries were sequenced on the HiSeq X platform (Illumina Inc.), generating 150 bp paired‐end reads across two lanes. Raw reads were deposited in the DDBJ database under the following accession numbers: BioProject PRJDB18203 (PSUB023205) and BioSample SAMD00789737‐SAMD00789985 (SSUB029785).

To detect SNPs and filter data, we first trimmed individual reads of bases after position 151 and removed low‐quality bases using Trimmomatic (Bolger, Lohse, and Usadel 2014). Next, we mapped the reads to the draft genome of *C. japonica* (Fujino et al. 2024) using BWA‐mem ver. 0.7.17 (Li and Durbin 2009) and detected polymorphisms using FreeBayes ver. 1.0.2 (Garrison and Marth 2012). These analyses were conducted using dDocent ver. 2.9.4 (Puritz, Hollenbeck, and Gold 2014), with default parameters for mapping and variant detection. The VCF files were filtered using VCFtools ver. 0.1.13 (Danecek et al. 2011), following the SNP filtering tutorial of the dDocent pipeline (https://www.ddocent.com/filtering/). The criteria for filtering included SNPs with more than 50% missing data across all individuals (‐‐max‐missing 0.5), sites with a minor allele count of at least 3 (‐‐mac 3), and a minimum mapping quality of 30 (‐‐minQ 30). We also considered allele balance at heterozygous loci using the VCF filter (AB > 0.25 & AB < 0.75 | AB < 0.01). Additionally, individuals with more than 10% missing data across all SNPs, as well as SNPs with a mean depth of less than 20 or a Minor Allele Frequency (MAF) of < 0.05, were excluded. On average, the percentage of missing genotypes per individual was 0.78% (ranging from 0.19% to 7.20%), indicating high‐quality genomic data across the analyzed samples.

### 2.2 Population Structure and Summary Statistics

Out of the 31,676 SNPs identified, 16,113 were used for genetic structure analysis after LD pruning. The pruning was conducted using PLINK ver.1.9 (Purcell et al. 2007) with a window size of 50 kb and a threshold for *r* ^2^ of 0.25 or below. To assess the genetic structure, PopCluster (Wang 2022) was utilized, considering a range of populations (*K*) from 1 to 10, with 10 iterations for each *K* value. Pairwise *F*_ST_ values between the genetic clusters detected by PopCluster were calculated to assess the genetic differentiation between clusters. Genetic diversity indices, such as observed and expected heterozygosity, inbreeding coefficient, allelic richness, and genetic differentiation between populations (both pairwise and overall *F*_ST_), were calculated using the R packages “hierfstat” (Goudet 2005) and “adegenet” (Jombart 2008; Jombart and Ahmed 2011). For the analysis of genetic diversity, populations with a smaller number of individuals from Yakushima and Shikoku, where the distances between populations were close, were combined and treated as a single population for each region. Next, AMOVA analysis was performed using the “poppr” (Kamvar, Tabima, and Grünwald 2014) package to evaluate the hierarchical structure of genetic diversity. Furthermore, genetic diversity and differentiation were also assessed for the climate‐associated loci identified in the following section.

### 2.3 Detection of Climate‐Associated Genes for Environmental Adaptation

To identify climate‐associated genes for environmental adaptation in *C. japonica* , we employed Latent Factor Mixed Models (LFMM: Frichot et al. 2013) as a univariate regression model, utilizing the R package “LEA” (Frichot and François 2015). LFMM performs multivariate linear regressions to evaluate the association between a response matrix, corresponding to SNP frequencies of individuals, and a matrix of environmental variables. We utilized a historical climate scenario (averages from 1990 to 2005) obtained from the Global Climate Model, MIROC6, downscaled to approximately 1‐km resolution in Japan (NIES2020 version 1.1; Ishizaki et al. 2022). From this climate scenario, we calculated the nineteen bioclimatic variables as outlined in WorldClim (Fick and Hijmans 2017) (Table S4). These variables were extracted for each individual using the “raster” package (Hijmans 2024) in R (R Core Team 2022). To mitigate the adverse effects of multicollinearity on our analysis, we conducted a Principal Component Analysis (PCA) on these 19 climatic variables. Prior to PCA, the climatic variables were scaled and centered to ensure that all variables contributed equally to the analysis and to align with the assumptions of linearity required for LFMM. By reducing the dataset to the first four principal axes, which explained 84.3% of the variance, we prepared our environmental data for inclusion in the LFMM analysis. The number of latent factors (*K*) to include in the LFMM model was chosen based on the results of the popCluster analysis previously described, ensuring an optimized representation of the underlying genetic structure. Before conducting the LFMM, missing values were imputed using allele frequencies from ancestral population estimates. These estimates were derived from the most reliable runs (i.e., those with the lowest cross‐entropy) for *K* = 4 using the “snmf” function in the “LEA” package. Multiple testing was controlled by converting the adjusted *p*‐values computed by LFMM into *q*‐values with the R package “qvalue” (Storey et al. 2024). Finally, we selected climate‐associated SNPs with FDR < 1% and median *z*‐scores exceeding the absolute value of 2. Following the identification of climate‐associated loci, we conducted functional annotation to provide insights into their relevance to climate adaptation in *C. japonica* . Genes located within 10 kb of the detected SNPs were identified based on their positions in the *C. japonica* draft genome. These genes were then functionally annotated by performing a BLAST search against the *Arabidopsis thaliana* gene set. Gene Ontology (GO) terms associated with the closest homologs were used to infer biological processes and pathways.

### 2.4 Geographic Distribution of Putative Adaptive Variation and Prediction of Genetic Offset

We used the Gradient Forest (GF) method (Ellis, Smith, and Pitcher 2012) to model adaptive genetic variation across environmental and spatial dimensions. This machine‐learning regression tree approach was originally designed to model the probability of species presence along environmental gradients and was instead applied to the distribution of alleles (Fitzpatrick and Keller 2015). In this study, we used the turnover functions in the gradient forest to identify environmental predictors that drive changes in allele frequencies across environmental gradients. Using the “gradientForest” package (Ellis, Smith, and Pitcher 2012), we fitted Gradient Forest models to environmental and spatial data for 22 populations comprising 249 individuals of *C. japonica* , with these data serving as predictors. The analysis focused on modeling the turnover of adaptive genetic variation across the landscape, treating the set of climate‐associated SNPs as the response variable. This approach allows the inference of key environmental drivers of observed shifts in genetic composition.

In our initial analysis, we evaluated 19 bioclimatic variables using the GF method, with the relative importance of each predictor variable indicated by weighted *R* ^2^ values (split importance; Ellis, Smith, and Pitcher 2012) To address multicollinearity among these variables, a Variance Inflation Factor (VIF) analysis was conducted using the “vifcor” function with a correlation threshold of 0.75 in the “usdm” package (Naimi et al. 2014) in R, resulting in the retention of 10 variables. Moreover, BIO13 and BIO11 were identified as critical and retained for their high explanatory power in the preliminary GF model and their significance for the adaptation of *C. japonica* , despite the removal of BIO4, which was highly correlated with BIO11. We also preserved BIO18 due to its representation of overall summer precipitation—an important environmental factor—and its inclusion was supported by the GF model's ability to accommodate correlated variables to some extent (Ellis, Smith, and Pitcher 2012). Ultimately, the GF modeling of adaptive genetic variation for *C. japonica* incorporated 11 bioclimatic variables: six related to temperature (BIO3, BIO5, BIO8, BIO9, BIO10, BIO11) and five related to precipitation (BIO13, BIO14, BIO15, BIO18, BIO19), presenting a balanced and comprehensive suite of environmental factors for our analysis.

To account for spatial variables, we used Principal Coordinates of Neighborhood Matrices (PCNMs) derived from the geographic coordinates of the sampled populations using the “pcnm” function in the “vegan” R package (Oksanen et al. 2023). This approach allowed spatial factors to be included in our analysis alongside the 11 selected climatic variables. Our model included two different sets of SNPs: (a) a set of neutral SNPs and (b) a set of SNPs significantly associated with climate variables. To visualize the results of the GF analysis, the dimensionality of the transformed environmental variables was reduced using principal component analysis (PCA), and the first three principal components (PCs) were assigned to a red‐green‐blue color palette. Procrustes rotation (Gower 1971) was applied to the PCA, mapping the predicted genetic composition of the neutral SNP dataset and the climate‐associated SNP dataset as described in Martins et al. (2018). The Procrustes residuals represent the absolute distance in genetic composition between the SNP datasets at each location, highlighting areas where adaptive genetic variation deviates from the background genetic variation.

To estimate genetic offsets under future climate scenarios, we projected allele frequencies using the climate scenario of MIROC6 under two socio‐economic pathways (SSP1‐2.6, SSP5‐8.5; NIES2020 version 1.1; Ishizaki et al. 2022) for 2030 (averaged from 2021 to 2040), 2050 (averaged from 2041 to 2060), and 2090 (averaged from 2081 to 2100). Genetic offsets were calculated as the Euclidean distance between current and future allele frequencies, indicating potential maladaptation due to climate change.

## 3 Results

### 3.1 Genetic Diversity and Genetic Structure

Table S1 presents the indicators of genetic diversity for each population based on LD‐pruned neutral SNPs (16,113 loci; hereafter referred to as neutral SNPs). The expected heterozygosity ranged from 0.256 to 0.289 with an average of 0.282, while the observed heterozygosity varied from 0.248 to 0.289, averaging 0.269. Allelic richness ranged from 1.254 to 1.288, with an average of 1.281. These results suggest higher genetic diversity in the western regions of Honshu (Figure S1). In contrast, genetic diversity decreased towards the northern part of the distribution, with lower values observed in Kyushu and Yakushima (Figure S1). The inbreeding coefficient (*F*_IS_) ranged from −0.007 to 0.070, with an average of 0.046, indicating no deviation from Hardy–Weinberg equilibrium in any population. Furthermore, no population‐specific private alleles were detected.

Table S1 also summarizes the genetic diversity indicators for climate‐associated SNPs (239 loci). The expected heterozygosity ranged from 0.255 to 0.391, averaging 0.325, while the observed heterozygosity varied from 0.235 to 0.349, with an average of 0.301. Allelic richness ranged from 1.461 to 1.691, with an average of 1.582. Similar to neutral SNPs, genetic diversity for climate‐associated SNPs was higher in the western regions of Honshu and decreased towards the northern regions. The inbreeding coefficient exhibited a broader range compared to neutral SNPs, from −0.050 to 0.135, with an average of 0.073. Overall, genetic diversity measures, including observed and expected heterozygosity, were higher for climate‐associated SNPs compared to neutral SNPs, indicating greater variability among adaptive loci. Measures of genetic differentiation, such as *F*_ST_ and Jost's *D*, were also higher for climate‐associated SNPs, reflecting stronger population differentiation among these loci (Table S2). Pairwise *F*_ST_ estimates among populations (Table S3) demonstrated higher genetic differentiation for climate‐associated SNPs compared to neutral SNPs. For example, populations in Yakushima (YKL, SRT, YKS) exhibited notably higher *F*_ST_ values for climate‐associated SNPs. Additionally, genetic differentiation was higher between populations from different genetic clusters, such as those located on the Sea of Japan side and the Pacific side.

The AMOVA analysis (Table 1) further highlighted differences in the partitioning of genetic variance between neutral and climate‐associated SNPs. For neutral SNPs, the majority of genetic variance was observed within populations (92.40%), while variance among clusters accounted for 4.48%. In contrast, for climate‐associated SNPs, variance among clusters was substantially higher (30.59%), while variance within populations accounted for 65.48%.

**TABLE 1:** Analysis of molecular variance (AMOVA) for *Cryptomeria japonica* using LD‐pruned neutral SNPs and climate‐associated SNPs.

Source of variation	Neutral SNPs				Adaptive SNPs		
	*df*	Sigma	% Variation	Phi	Sigma	% Variation	Phi
Among clusters	3	114.526	4.48	PhiCT = 0.045	19.390	30.59	PhiCT = 0.306
Among populations within clusters	18	79.862	3.12	PhiSC = 0.033	2.492	3.93	PhiSC = 0.057
Within populations	227	2364.324	92.40	—	41.512	65.48	—
Total	248	2558.713	100	PhiST = 0.076	63.394	100	PhiST = 0.345

PopCluster analysis identified four clusters, which is consistent with previous reports (Kimura et al. 2014; Yoshihiko Tsumura et al. 2014; Uchiyama et al. 2014). These clusters were distinctly separated into the northern tip of the Sea of Japan side, the southern tip of Yakushima, and the Pacific and Sea of Japan sides of Honshu (refer to Figure 1). However, populations in Shikoku showed a mixture of Pacific and Sea of Japan side clusters within the population. These results were identical to those obtained using the snmf function in the R package LEA (Frichot and François 2015). Therefore, *K* = 4 was used as the latent factor for LFMM analysis. Pairwise *F*_ST_ values between the genetic clusters of *C. japonica* showed the lowest differentiation between the Northern (Tohoku) and Japan Sea clusters (0.0244), while the highest differentiation was observed between the Northern (Tohoku) and Southern (Yakushima) clusters (0.0958). Within‐cluster differentiation, represented by diagonal values, was highest in the Southern (Yakushima) cluster (0.0633). These results are summarized in Table 2.

**TABLE 2:** Pairwise *F*_ST_ values between genetic clusters of *Cryptomeria japonica* .

	Northern (Tohoku)	Japan Sea	Pacific Ocean	Southern (Yakushima)
Northern (Tohoku)	*0.0455*			
Japan Sea	0.0244	*0.0296*		
Pacific Ocean	0.0491	0.0313	*0.0339*	
Southern (Yakushima)	0.0958	0.0773	0.0694	*0.0633*

*Note:* The values along the diagonal, shown in italics, indicate the degree of genetic differentiation of each cluster relative to the overall population, including the cluster itself.

### 3.2 Identifying Putatively Adaptive Variants and Their Geographical Distribution

**FIGURE 2:**
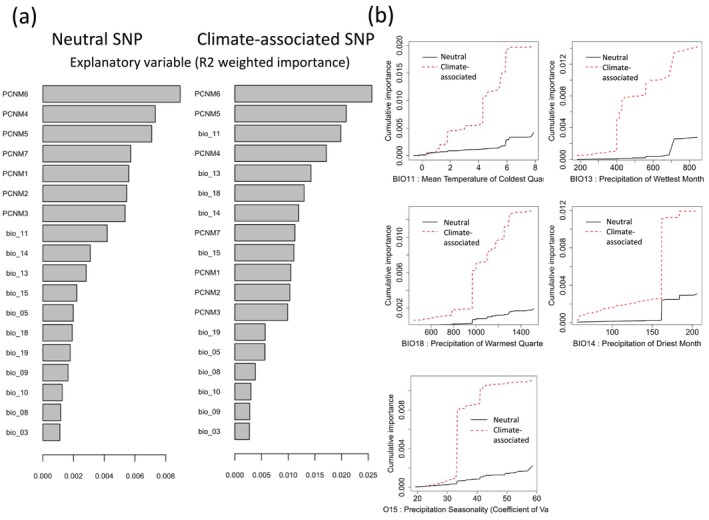
Comparison of explanatory variables for Neutral SNPs and Climate‐associated SNPs and cumulative importance of environmental gradients based on Gradient Forest modeling. (a) The *R* ^2^ weighted importance of explanatory variables ranked for both Neutral SNPs (left) and Climate‐associated SNPs (right). PCNMs represent spatial autocorrelation variables, while BIO variables represent climatic factors. The variables are arranged in descending order of their explanatory power. (b) Cumulative importance of allelic turnover along environmental gradients for both Neutral SNP (black solid line) and Climate‐associated SNP (red dashed line) models. Significant differences were observed between Neutral and Climate‐associated models across the top five variables: BIO11 (Mean Temperature of Coldest Quarter), BIO13 (Precipitation of Wettest Month), BIO18 (Precipitation of Warmest Quarter), BIO14 (Precipitation of Driest Month), and BIO15 (Precipitation Seasonality).

The LFMM analysis identified 240 significant correlations (FDR < 0.01) out of 31,676 SNPs when evaluated against four principal components derived from climatic variables (Figure S3). After removing one overlapping locus, a total of 239 loci were classified as adaptive variations. These loci were further examined using Gradient Forest (GF) analysis, which incorporated 11 environmental variables and six Principal Coordinates of Neighbor Matrices (PCNM). Among these, BIO11 (Mean Temperature of the Coldest Quarter) emerged as the most influential variable, followed by BIO13 (Precipitation of the Wettest Month), BIO18 (Precipitation of the Warmest Quarter), BIO14 (Precipitation of the Driest Month), and BIO15 (Precipitation Seasonality) (Figure 2a).

The cumulative importance of allelic turnover along these environmental gradients was compared between adaptive and neutral SNP models. Notable differences were observed, with steep changes in allele frequencies occurring at specific environmental thresholds—such as 4°C and 6°C for BIO11, around 400 mm for BIO13, near 1000 mm for BIO18, around 160 mm for BIO14, and approximately 32 for BIO15. These thresholds suggest that specific environmental conditions may exert strong selective pressures on the adaptive loci (Figure 2b). Further details on the variables used in the GF model are provided in Figure S4.

**FIGURE 3:**
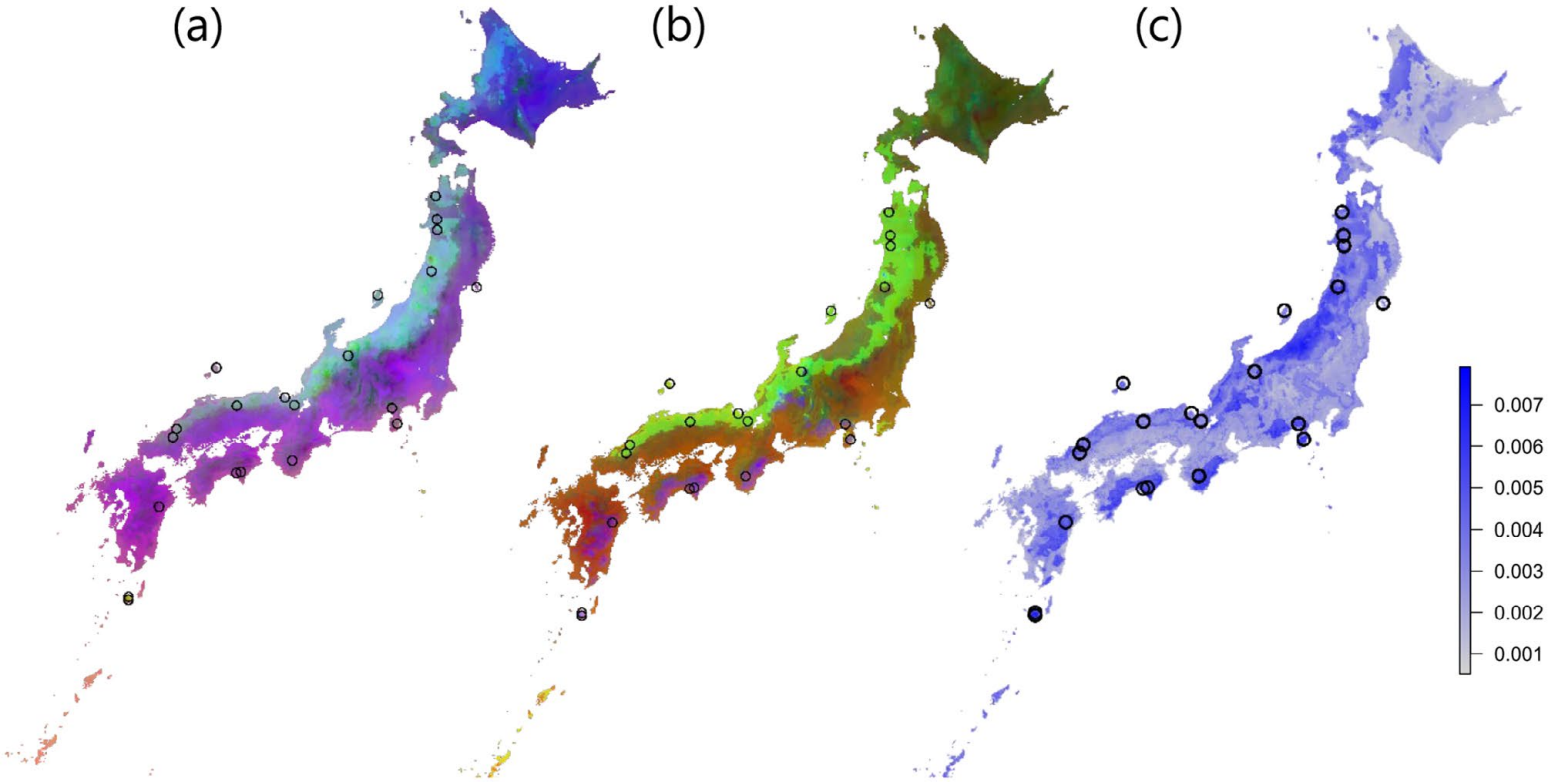
Spatial distribution of allelic composition predicted by the Gradient Forest model. (a) Results for neutral SNPs and (b) results for climate‐associated (adaptive) SNPs. The maps were generated using the first three principal components (PCs) from the PCA. Regions with similar colors are genetically closer, indicating a high degree of genetic similarity. (c) Differences between neutral and adaptive SNP compositions evaluated using Procrustes rotation. Darker blue regions represent areas where the compositions of neutral and adaptive SNPs differ more substantially. Sampling sites are marked with black circles.

Spatial genetic structure for both neutral and adaptive SNP sets was also evaluated across Japan. A division between populations on the Sea of Japan side and those on the Pacific Ocean side was evident in both SNP sets (Figure 3a,b). However, structural differences between the two sets, highlighted by Procrustes residuals, were particularly pronounced in regions with high rainfall on the Pacific side and in snowy areas along the Sea of Japan side (Figure 3c). These findings underscore the localized impacts of environmental gradients on genetic architecture. From the 239 adaptive SNPs identified, a total of 94 genes were located within 10 kb of these SNPs based on their positions in the *C. japonica* draft genome. Of these, 72 genes were successfully matched to homologous genes in *Arabidopsis thaliana* through BLAST analysis. Among the matched genes, 38 were annotated with functional notes based on their Gene Ontology (GO) terms and known roles in biological processes. The results are comprehensively summarized in Table S5, which includes details on GO terms, functional roles, and their relevance to climate adaptation.

### 3.3 Predicting Genetic Offset

**FIGURE 4:**
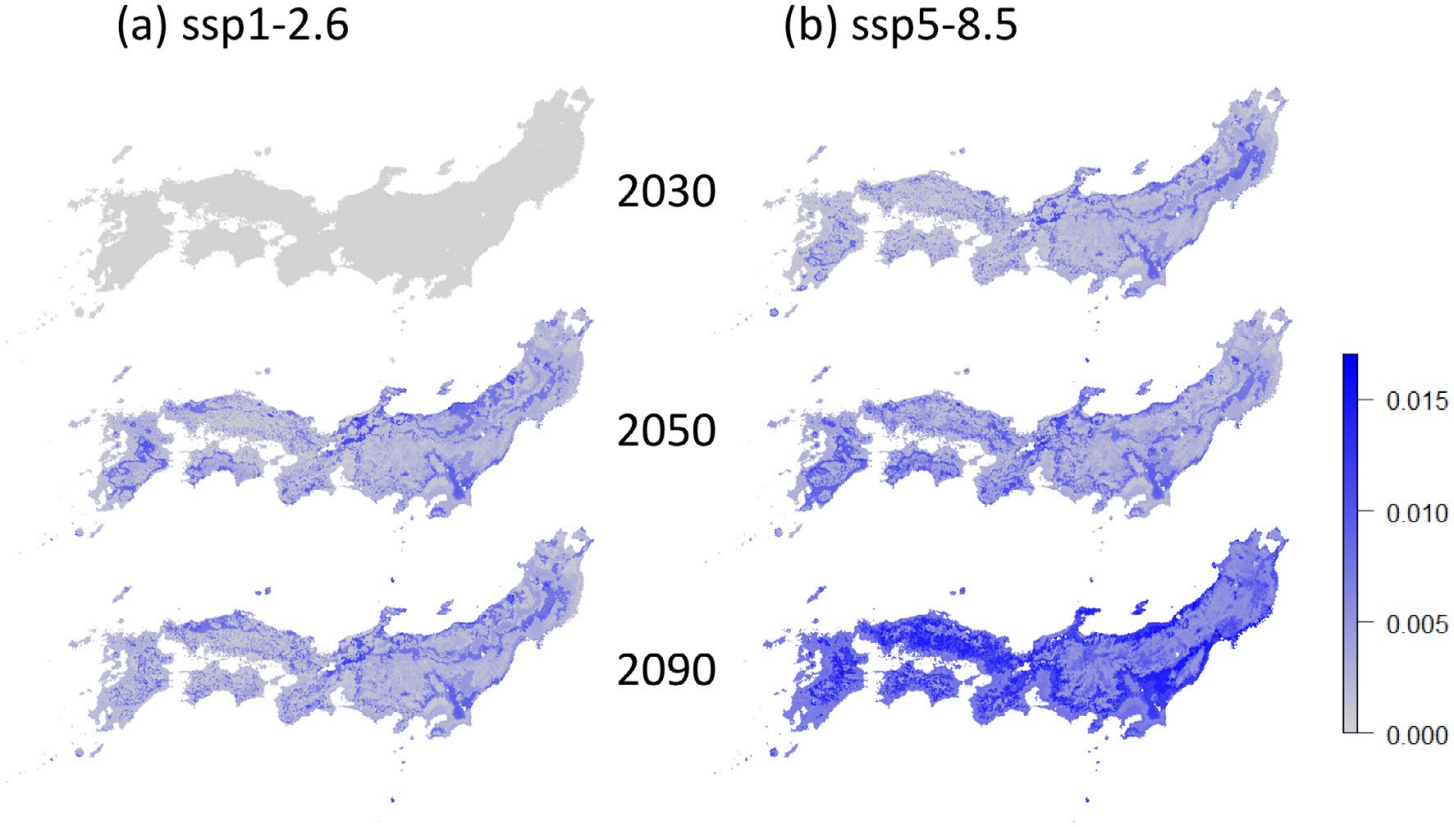
Projected genetic offset for *Cryptomeria japonica* under SSP 126 and SSP 585 scenarios. This set of maps represents the geographic distribution of predicted genetic offset for three time points: 2030, 2050, and 2090. Panel (a) corresponds to projections under the SSP 126 scenario, while panel (b) is under the SSP 585 scenario. In each map, a blue scale is used, with lighter colors (light grey) indicating minimal genetic divergence from current conditions and darker blue tones representing greater genetic offset. The progression from lighter to darker blue illustrates the increasing impact of climate change on the genetic composition of *C. japonica* populations over time. The maps for 2090, particularly under SSP 585, highlight regions with the most significant evolutionary pressures due to climate change, as predicted by Euclidean distances between current and future allele frequencies.

The concept of genetic offset, which quantifies the cumulative divergence between current allele frequencies and those required under future climatic conditions, was used to evaluate potential impacts of climate change. Predictions were made for 2030, 2050, and 2090 under two socio‐economic scenarios: SSP 1–2.6 and SSP 5–8.5 (Figure 4). A higher genetic offset suggests greater potential for reduced fitness due to environmental mismatch.

The results indicate that areas with high genetic offset are expected to expand over time under both scenarios. Under the SSP 1–2.6 scenario, genetic offset showed modest increases, with some regions, such as inland Kyushu, even exhibiting a reduction by 2090. In contrast, the SSP 5–8.5 scenario predicted substantial expansions of high‐offset areas by 2090, particularly in the Chugoku region of western Japan, coastal lowlands along the Sea of Japan, and inland areas on the Pacific side of eastern Japan. Conversely, high‐altitude areas in eastern and northern Japan showed relatively smaller increases in genetic offset, indicating potential resilience to warming in these regions.

## 4 Discussion

This study provides a comprehensive analysis of the genetic diversity and adaptive genetic structure of *C. japonica* , employing integrative population genetics and landscape genomics approaches. By integrating genetic differentiation analysis, climate‐association models, and future climate scenario projections, we identified key climatic factors such as winter temperature, summer precipitation, and snowfall that drive adaptive genetic variation in this species. The findings highlight the role of historical processes and regional environmental heterogeneity in shaping genetic structure and adaptive potential across its natural range. Furthermore, predictions of genetic offset under future climate scenarios underscore the challenges posed by climate change to forest management and the need for adaptive strategies. These insights not only enhance our understanding of the evolutionary dynamics of *C. japonica* but also provide valuable guidance for conservation and sustainable forestry management in the face of global warming.

### 4.1 Genetic Diversity and Genetic Structure

The analysis of 16,113 independently LD‐pruned SNPs revealed a genetic differentiation of 0.0354 between populations, which was slightly lower but statistically significant (*p* < 0.05) compared to the 0.0506 reported by Tsumura et al. (2014) using SNPs within exons. The distribution of the four genetic clusters detected by the popCluster analysis was consistent with previous reports, showing a major division between the Pacific Ocean side and the Sea of Japan side, with additional separations at the northernmost and southernmost distributions (Figure S2). Except for the cluster at the southernmost distribution in Yakushima, the other three clusters exhibited individual‐level admixture in some populations, suggesting ongoing gene flow. These findings are further supported by the pairwise *F*_ST_ values calculated between the four genetic clusters. The low differentiation between the Northern (Tohoku) and Japan Sea clusters (*F*_ST_ = 0.0244) reflects a relatively high degree of genetic connectivity, likely facilitated by shared historical migration or gene flow along the Sea of Japan coastline. Conversely, the higher differentiation observed between the Northern (Tohoku) and Southern (Yakushima) clusters (*F*_ST_ = 0.0958) suggests significant genetic isolation, which may be attributed to the geographic and ecological distance between these regions. The within‐cluster differentiation, as indicated by diagonal *F*_ST_ values, was particularly high in the Southern (Yakushima) cluster (*F*_ST_ = 0.0633). This could reflect local adaptation or historical isolation due to the island's unique climatic and ecological conditions.

Genetic diversity among natural populations of *C. japonica* , as measured by expected heterozygosity and allelic richness, was notably high in the Chugoku region of western Japan (Figure S1). This area has been inferred from pollen analysis and species distribution modeling to have harbored extensive *Cryptomeria* forests during the Last Glacial Maximum (Kimura et al. 2014; Tsukada 1982). Moreover, the current warm and humid climate of this region is considered ideal for *C. japonica* , suggesting that stable conditions throughout glacial and interglacial periods have contributed to the high genetic diversity observed. Among the populations showing high genetic diversity within the Japan Sea cluster, the southernmost population on Kyushu Island (ONN) exhibited lower genetic diversity. This population, located in the high‐altitude areas of Oninome Mountain, represents the only natural forest on Kyushu Island and is thought to have experienced reduced genetic diversity due to small population size. Furthermore, its expansion continues to be restricted by the surrounding warm‐temperate vegetation (Tsukada 1982).

Conversely, many populations in the Pacific cluster exhibited low genetic diversity. Multiple refugia for *C. japonica* on the southern coastline of the Pacific side have been reported through pollen analysis and genetic studies (Takahashi et al. 2005; Tsukada 1982). However, these populations are believed to have undergone substantial reductions in size during the ice ages (Tsukada 1982), which likely contributed to the decrease in genetic diversity. The populations in Shikoku, at the southern end of the Pacific cluster, maintain high genetic diversity, and a notable feature of this population is the mixing of genetic lineages from both the Pacific and Japan Sea clusters (Figure 1). This admixture may have enhanced their diversity. The northern end of the Pacific side also maintains high diversity, although the reasons remain unclear. Notably, slightly north inland, regions have been identified with unique genetic clusters in other species (Tsuda et al. 2015), considered to have been refugia during the ice ages. It is possible that *C. japonica* also had relatively stable refugia in these areas.

However, populations belonging to clusters identified at both the southern and northern edge of *Cryptomeria* distribution exhibited low genetic diversity. The northern genetic cluster is thought to be survivors from cryptic refugia during the Last Glacial Maximum, as supported by previous genetic studies and species distribution modeling results (Kimura et al. 2014). While secondary contact and mixing with more southern groups appear to be progressing, the diversity of these populations has not fully recovered. The southernmost Yakushima population is isolated by a strait, forming a distinct cluster. Limited natural *Cryptomeria* forests on Kyushu Island and ongoing isolation from Honshu populations are thought to have led to a reduction in diversity due to genetic drift. In this study, to avoid type I errors during association analysis, loci with a minor allele frequency (MAF) < 0.05 were excluded from the analysis, thus previously detected rare genetic diversities in refugia through SSR analysis (Takahashi et al. 2005) have not been evaluated.

### 4.2 Geographical Distribution of Putative Adaptive Genetic Variation

The analysis of genetic diversity in climate‐associated SNPs revealed notable differences in diversity patterns compared to neutral SNPs. While adaptive SNPs showed lower diversity in northern populations, consistent with the patterns observed in neutral SNPs, the southern populations, including those in Kyushu and Yakushima, maintained relatively high adaptive diversity. This finding suggests that directional selection may be acting on adaptive loci in the colder northern environments, potentially reducing diversity in response to environmental pressures. However, it is important to note that these results represent averages across 239 loci significantly associated with four principal components derived from 19 climatic variables. The specific allele frequency patterns and their associated environmental gradients likely vary across loci, warranting further examination within the GF model results presented later in this section.

Moreover, the results of AMOVA and genetic differentiation metrics further support the role of environmental differences in shaping adaptive genetic structure. In particular, higher genetic differentiation for adaptive loci, especially among genetic clusters from distinct environmental regions such as the Pacific and Japan Sea sides, highlights the influence of divergent selection pressures in driving local adaptation. These patterns underscore the importance of environmental heterogeneity in maintaining genetic differentiation in *C. japonica* populations. These findings provide a broader context for understanding the patterns of adaptive diversity and differentiation, which are further explored in relation to specific environmental gradients in the following sections.

The investigation into the geographical distribution of putative adaptive variation is pivotal in understanding how genetic diversity correlates with environmental gradients and could potentially inform conservation strategies for species facing climate change. The results of the GF analysis for the 239 climate‐associated loci indicated that the most influential factor was the mean temperature of the coldest quarter (BIO11) (Figure 2). The areas where steep changes in allele frequencies were observed for BIO11 spanned a broad region from coastal lowlands to high‐elevation inland areas across the archipelago (Figure S5). Notably, the boundary between the northern glacial cryptic refugia‐origin populations and other populations around a BIO11 value of approximately 0.5°C showed almost no allelic frequency changes, suggesting that the detected adaptation to BIO11 is not indicative of adaptation to the colder environments in the northernmost regions. BIO11 was highly correlated with the excluded variables BIO1, BIO2, BIO4, BIO6, and BIO7 from the VIF analysis, indicating that it encompasses the temperature variations between summer and winter. Plant phenology is mainly influenced by day length and temperature. In *C. japonica* , growth is suppressed under short‐day and low temperatures. Analyses of annual transcriptome variations under controlled environments and in the field have shown that the interaction between day length and temperature regulates the annual transcriptome dynamics (Nose et al. 2020). This suggests that in *C. japonica* , temperature could be a crucial factor determining the phenology of the growth/dormant periods at locations with the same day length (latitude). In evergreen trees like *C. japonica* , cold tolerance is acquired during winter. However, during late autumn and early spring, when cold tolerance is insufficient, temperatures and frosts that are not problematic in winter can cause significant damage. In *C. japonica* , it has been reported that osmotic potential, an indicator of leaf solute concentration, and water potential at the point of turgor loss decrease from autumn to winter, showing a sharp decline, especially at temperatures below 5°C (Norisada et al. 2005). The current results suggest that the patterns observed may capture genetic adaptations related to low winter temperatures across the entire distribution range of *C. japonica* .

The next most important variables, BIO13 and BIO18, which relate to summer precipitation. The rainy season in Japan starts in June, increasing rainfall across the country except in Hokkaido and parts of the Seto Inland Sea region, particularly along the Pacific Ocean side of western Japan, Kyushu, and parts of the Sea of Japan side in eastern Japan, where monthly rainfall can exceed 500 mm (Figure S5). The areas where allelic turnover steeply changed in BIO13 and BIO18 were roughly the same and centered around regions with high summer rainfall.

The fourth most important factor was BIO14 (precipitation of the driest month), which corresponds to the driest month in winter in Japan. The high BIO14 areas were scattered along the Sea of Japan, extending discontinuously to the northern distribution limits. These areas overlapped with regions ranked sixth in importance, BIO19 (precipitation of the coldest quarter), indicating potential adaptation to winter precipitation (snowfall). In addition to environmental factors, the high ranking of spatial variables (PCNMs) reflects the effects of isolation by distance but may also suggest the importance of unmeasured environmental predictors not included in the model. Additionally, QTL analyses conducted in *Cryptomeria* provenance trials have suggested that short periods of drought in early spring significantly influence growth performance (Mori et al. 2020). This indicates the need to consider factors beyond temperature and precipitation, which were used in the current study.

The differences in genetic structure between neutral and climate‐associated loci sets are visualized in Figure 3c using Procrustes residuals, with darker colors indicating greater differences. This pattern likely reflects the intensity of adaptation occurring at each location. Strong adaptation in *C. japonica* was detected across extensive regions, including snowy areas on the Sea of Japan side, colder northern regions, high rainfall areas on the southern Pacific side, and some inland areas. The Sea of Japan side of the Japanese archipelago is known for its rare heavy snowfall areas globally, with annual snowfall depths exceeding several meters. This phenomenon occurs as moist air from the warm currents flowing from the south in the Sea of Japan is transported by seasonal winds from Siberia and causes heavy snowfall when it hits the high mountains of the Japanese archipelago. This heavy snowfall environment creates intra‐species variation in various species, serving as a strong natural selection pressure for plants in the region (Nagamitsu et al. 2014; Sakai 1976; Uemura 1989). In *C. japonica* , variation in leaf morphology has led to the classification of some populations on the Sea of Japan side as a distinct variety, *C. japonica* (L. fil.) D. Don var. *radicans* Nakai. Additionally, genetic differentiation has been observed between populations on the Sea of Japan side and those on the Pacific side (Tsumura 2023). In an example of survival and growth investigations through reciprocal transplanting of plus trees, individuals selected in low snowfall areas showed decreased survival and growth in heavy snowfall environments, indicating an inability to adapt to snowfall (Miura et al. 2009). The current results, showing structural variations in adaptive genetic variations in heavy snowfall areas, suggest that the observed phenomenon indicates *C. japonica* 's adaptation to snow conditions.

Interestingly, the analysis also revealed strong adaptation in areas with high summer rainfall. In addition to Yakushima Island at the southernmost tip of the mainland, regions in the southern part of the mainland with more than 1000 mm of summer rainfall suggest strong environmental stress even for *C. japonica* , which prefers moist conditions. Although high precipitation is unlikely to be a direct stressor in itself, it is thought to indirectly generate a variety of environmental stresses. One possibility is that the high temperature and humidity conditions in these regions increase the likelihood of infection by fungi and viruses or insect damage. Indeed, it has been reported that variations in terpenes stored in and emitted from leaves of *C. japonica* are significantly correlated with the composition of antagonistic fungal species inhabiting the cedar (Hiura et al. 2021). Additionally, gene expression analysis conducted at a provenance trial site on Kyushu Island has demonstrated that seedlings originating from high precipitation areas on the Pacific side exhibit significantly elevated expression of defense response genes during the summer compared to seedlings from other regions (Ujino‐Ihara et al. unpublished data).

Japan's maritime climate, situated in proximity to the ocean, results in high annual rainfall, ranking it among the rainiest regions globally. However, the seasonal patterns of rainfall exhibit considerable variability across different regions, with notable fluctuations. The spatiotemporal variations in rainfall are likely to contribute significantly to the natural selection pressures on *C. japonica* .

### 4.3 Genetic Offset of *Cryptomeria japonica* Under Future Climate Scenarios

Using Gradient Forest (GF) modeling, we estimated the potential genetic offset of *C. japonica* under future climate scenarios based on current gene–environment associations. Under the SSP1‐2.6 scenario, genetic offset of *C. japonica* increased towards 2050 in parts of eastern Japan, particularly in the Kanto region, lowland areas along the Sea of Japan, and certain inland regions nationwide. However, no significant increase in offset was observed towards 2090 (Figure 4). This suggests that a temperature rise of around SSP1‐2.6 may not lead to a substantial increase in genetic offset for *Cryptomeria* across Japan. Notably, areas in inland Kyushu where high offsets were detected in 2050 had diminished by 2090, potentially influenced by predictions indicating that the increased summer precipitation in 2050 would decrease back to current levels by 2090.

Global warming is expected to cause widespread drying and increased drought frequency in regions such as Eurasia and the Americas (Chiang, Mazdiyasni, and AghaKouchak 2021; Spinoni et al. 2021), raising concerns about significant impacts on forest trees (Allen et al. 2010; Jing et al. 2022). In contrast, Japan's maritime climate predicts relatively stable annual precipitation totals even post‐warming, suggesting that large‐scale drying may not occur as in those regions. However, the frequency and intensity of extreme precipitation events are expected to increase (Higashino, Hayashi, and Aso 2022). These changes in rainfall patterns may alter the optimal allele frequencies for precipitation‐related adaptive genetic variations in *Cryptomeria*, thereby affecting the genetic offset.

Under the SSP5‐8.5 scenario, an increase in offset was observed from as early as 2030, with significant rises predicted by 2090 in broad areas of western Japan and lowland eastern Japan. Despite relatively stable total summer and winter precipitation predicted under the SSP5‐8.5 scenario, the substantial increase in offset across these regions is likely driven by rising temperatures. The most critical climate factor identified by the GF model was the mean temperature of the coldest quarter (BIO11), with allelic turnover occurring around 2°C, 4°C, and 6°C, spanning a wide range from lowlands to high inland areas across the long Japanese archipelago. For instance, in the Chugoku region, a center of *Cryptomeria* distribution and high genomic diversity, offset rose sharply as the mean winter temperature increased from around 4°C–8°C by 2090 under SSP5‐8.5. Although BIO11 correlates strongly with several other temperature variables, suggesting it represents more than just winter temperature, these findings indicate a broad increase in offset at average winter temperatures around 4°C. Physiological and ecological studies also predict adverse effects of warming on *Cryptomeria* growth. Shigenaga et al. (2005) predicted that increased evapotranspiration would reduce water‐use efficiency, increasing growth stress in low‐precipitation areas like the Kanto Plain and Setouchi region. Furthermore, growth data from *Cryptomeria* plantations predict a decline in net primary production during summer in southwestern Japan, including Kyushu, Shikoku, and the Chugoku region, post‐warming (Toriyama et al. 2021). While the genetic data‐based predictions from this study do not pinpoint specific mechanisms of maladaptation, the agreement with results from other methodologies underscores their significance.

The approach employed in this study—spatial modeling of genome‐wide DNA markers and climatic variables to predict allele frequency changes and genomic offset under future climate scenarios—has garnered significant attention in recent years. This method is particularly valuable for forecasting tree species' responses to future climates, especially in situations where transplant experiments are logistically challenging or impractical. However, the validation of whether predicted offsets accurately correspond to actual fitness declines remains an ongoing challenge (Fitzpatrick et al. 2021; Lotterhos 2024). Studies integrating common garden phenotypic measurements with genomic offset predictions have demonstrated that these predictions can closely correspond with observed declines in fitness (Fitzpatrick et al. 2021; Lind et al. 2024). Nonetheless, the lack of rigorous study designs for thorough validation has been highlighted (Lotterhos 2024). Furthermore, simulation studies suggest that the predictive accuracy of genomic offset methods is significantly influenced by the degree of local adaptation and tends to decline when applied to novel environments that markedly differ from the conditions used for model training (Lind and Lotterhos 2024; Lotterhos 2024). Despite these limitations, genomic offset modeling remains an indispensable tool for tree species, particularly in cases where experimental approaches are impractical. With the ongoing expansion of both theoretical frameworks (Gain et al. 2023) and empirical validation efforts, this methodology holds considerable promise for advancing our understanding of how trees respond to environmental changes.

## 5 Conclusion and Implications for *Cryptomeria* Management and Limitations of Predictions

This study elucidated the geographical distribution of environmental adaptive genetic variations in *C. japonica* , Japan's most crucial forestry species, and identified key climatic factors influencing these variations. Winter temperature, summer precipitation, and winter snowfall emerged as significant selective pressures. The four genetic lineages detected by neutral genetic loci are thought to have formed through isolation and distribution expansion cycles during glacial and interglacial periods. These lineages are distributed under distinct climatic conditions, indicating significant differentiation in adaptive variation among them. Such differentiation underscores the importance of treating these lineages as conservation units, not only for in situ preservation but also as a basis for delineating forestry seed zones. The movement of planting stock between these lineages poses a significant risk of reducing fitness due to maladaptation.

At the same time, the structure of adaptive variation observed within each lineage indicates that allele frequencies shift significantly along local environmental gradients. This suggests that even within the established lineages, finer‐scale management practices may be necessary to account for local adaptation.

Currently, *C. japonica* is conserved in situ across 33 genetic resource conservation forests in regions such as Kyushu and the Chugoku area. However, as global warming progresses, certain populations may become increasingly difficult to maintain under in situ conditions. For these populations, ex‐situ conservation should be considered as a complementary strategy to ensure the preservation of genetic diversity. Furthermore, genetic analyses of breeding populations have revealed that in areas like the Chugoku and Kyushu regions, the stock used in plantations often belongs to genetic lineages distinct from those of natural forests (Uchiyama et al. 2014). This discrepancy raises questions about whether the projected impacts on natural forests will directly translate to the widely used plantation Cryptomeria, highlighting the need for further investigation. Nevertheless, in regions with high predicted genetic offsets, careful selection of planting stock remains critical to mitigate the risks of maladaptation under future climatic conditions.

The GF modeling results revealed aspects of *Cryptomeria*'s environmental adaptation and predicted fitness declines under future climate scenarios. However, several limitations already noted in previous studies apply (Lind and Lotterhos 2024; Rellstab, Dauphin, and Exposito‐Alonso 2021). These include assumptions about the equilibrium of current adaptive genetic variation with the climate, the lack of consideration for gene flow, and the absence of certain environmental factors in the model. Furthermore, adaptation is influenced by complex processes such as migration, mutation, and effective population size, making actual evolutionary responses to climate change more intricate than modeled predictions (Fitzpatrick and Keller 2015). Nonetheless, mitigating climate change impacts on this keystone species remains a critical priority. Future research should validate model predictions through provenance trials, incorporate additional environmental factors, and refine the understanding of adaptive genetic variations at finer spatial scales. Such efforts will strengthen conservation strategies and forest management plans, ensuring the long‐term resilience of *Cryptomeria* forests.

This study represents the first investigation into tree species adaptation in the Japanese archipelago through spatial modeling of adaptive genetic variation. Continued research across diverse tree species will advance the understanding of plant adaptation in Japan and inform strategies to maintain resilient forest ecosystems in an era of rapid environmental change.

## Conflicts of Interest

The authors declare no conflicts of interest.

## Supporting information


Figures S1–S5

Tables S1–S5

## Data Availability

Raw GBS data are available on DDBJ's Sequence Read Archive (DRA) as ‘BioProject’ PRJDB18203 and ‘BioSamples’ SAMD00789737–SAMD00789985 (SSUB029785).
