# Supplementary material for: Climate‐Associated Genetic Variation and Projected Genetic Offsets for Cryptomeria japonica D. Don Under Future Climate Scenarios
Source: Evol Appl. 2025 Feb 6;18(2):e70077. doi: 10.1111/eva.70077 (PMC11802333; doi:10.1111/eva.70077)
Supplement: Supplementary file 1 — Figures S1–S5 [file EVA-18-e70077-s002.pptx]

## Slide 1
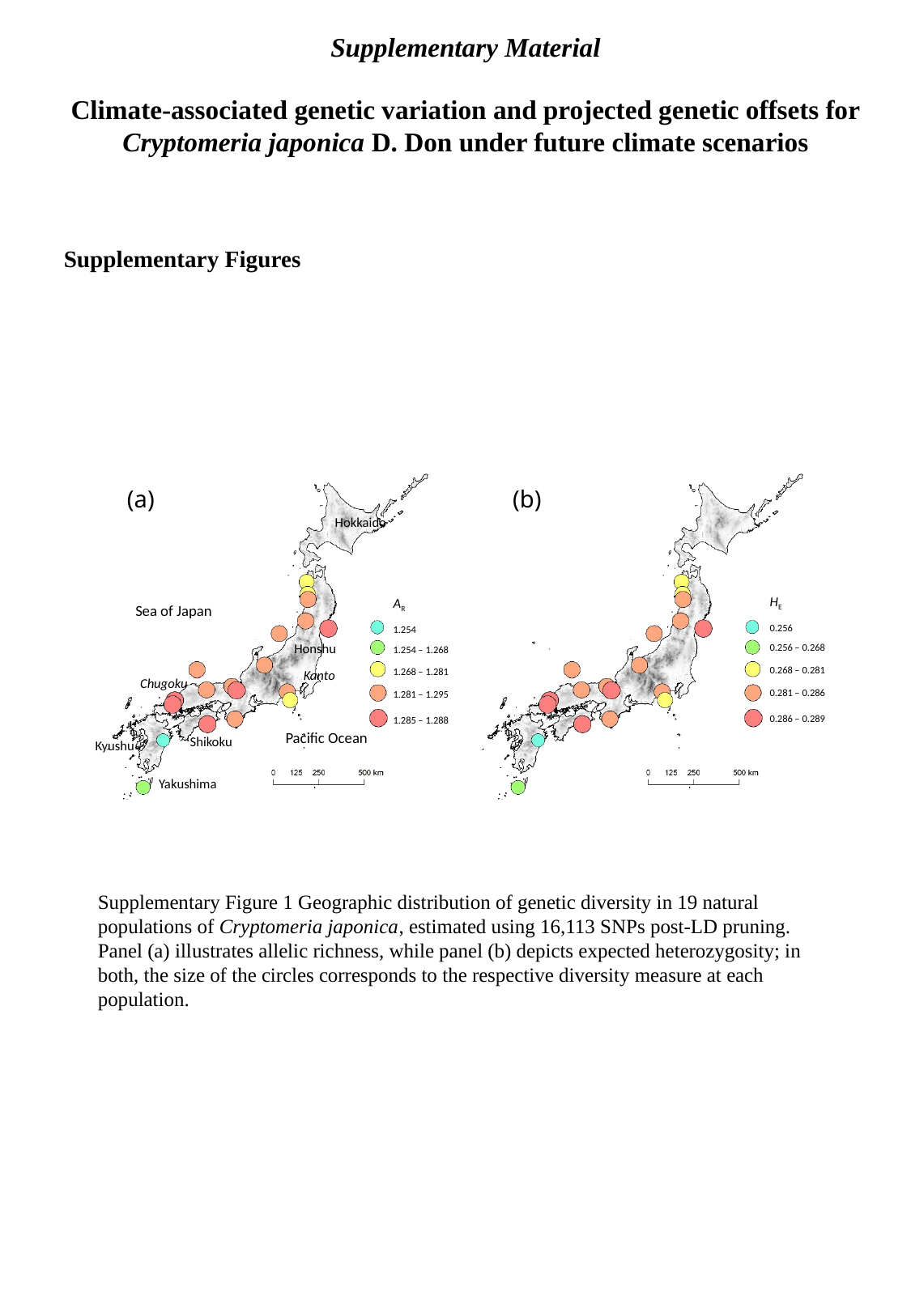

Supplementary Material
Climate-associated genetic variation and projected genetic offsets for Cryptomeria japonica D. Don under future climate scenarios
Supplementary Figures
(a) (b)
Hokkaido
HE
0.256
0.256 – 0.268
0.268 – 0.281
0.281 – 0.286
0.286 – 0.289
AR
1.254
1.254 – 1.268
1.268 – 1.281
1.281 – 1.295
1.285 – 1.288
Sea of Japan
Honshu
Kanto
Chugoku
Pacific Ocean
Shikoku
Kyushu
Yakushima
Supplementary Figure 1 Geographic distribution of genetic diversity in 19 natural populations of Cryptomeria japonica, estimated using 16,113 SNPs post-LD pruning. Panel (a) illustrates allelic richness, while panel (b) depicts expected heterozygosity; in both, the size of the circles corresponds to the respective diversity measure at each population.

## Slide 2
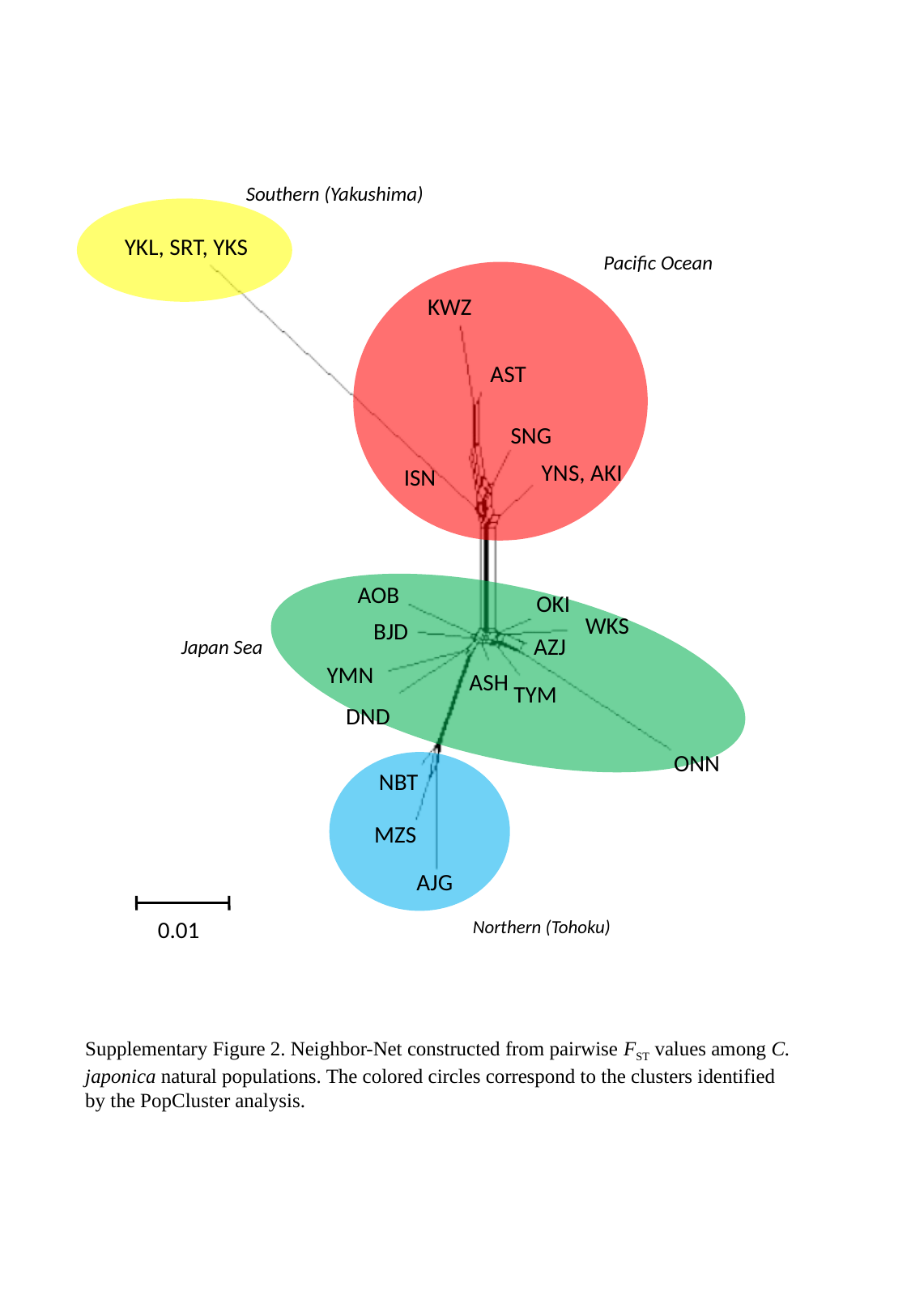

Southern (Yakushima)
YKL, SRT, YKS
Pacific Ocean
KWZ
AST
SNG
YNS, AKI
ISN
AOB
OKI
WKS
BJD
Japan Sea
AZJ
YMN
ASH
TYM
DND
ONN
NBT
MZS
AJG
0.01
Northern (Tohoku)
Supplementary Figure 2. Neighbor-Net constructed from pairwise FST values among C. japonica natural populations. The colored circles correspond to the clusters identified by the PopCluster analysis.

## Slide 3
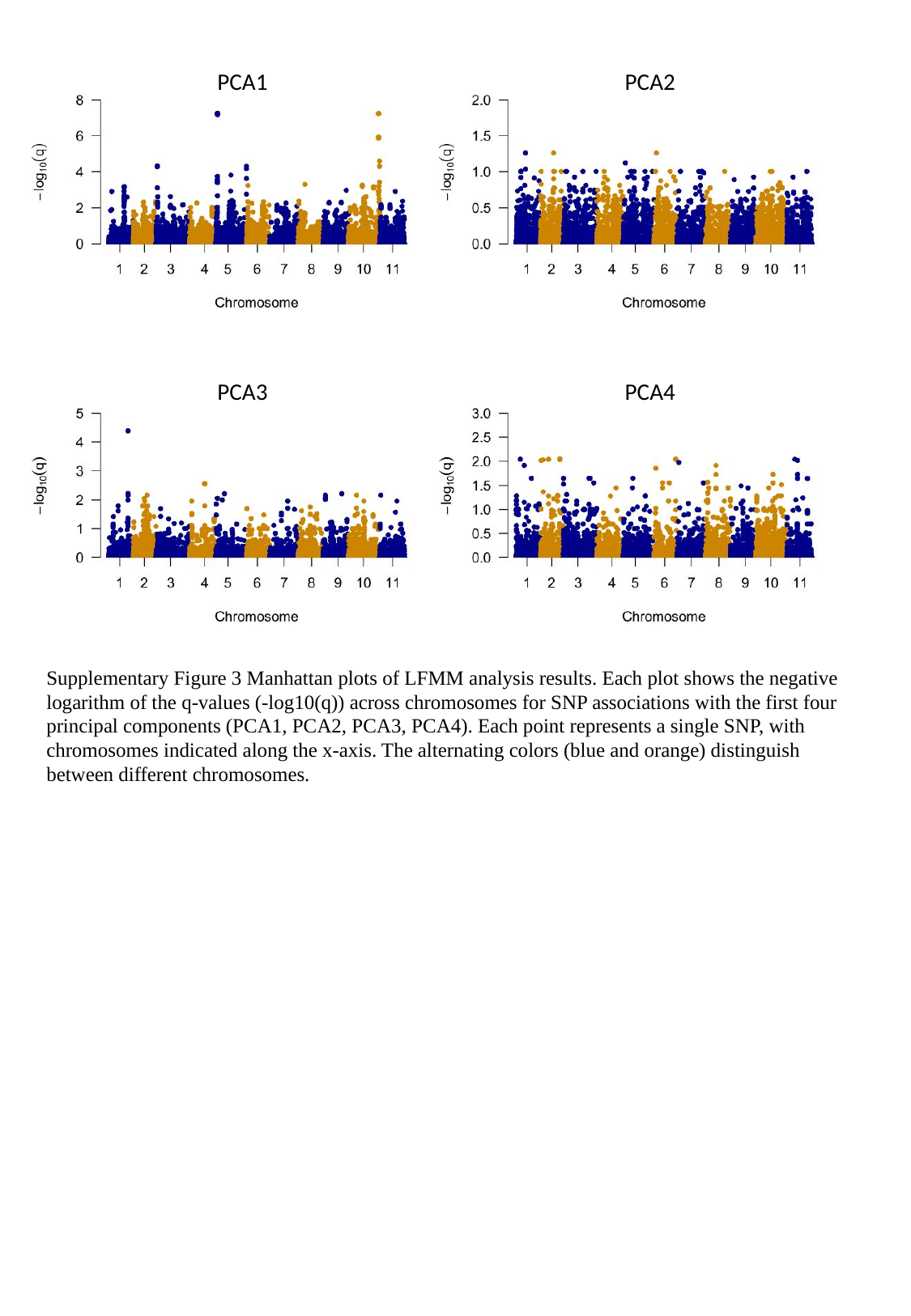

PCA1 PCA2
PCA3 PCA4
Supplementary Figure 3 Manhattan plots of LFMM analysis results. Each plot shows the negative logarithm of the q-values (-log10(q)) across chromosomes for SNP associations with the first four principal components (PCA1, PCA2, PCA3, PCA4). Each point represents a single SNP, with chromosomes indicated along the x-axis. The alternating colors (blue and orange) distinguish between different chromosomes.

## Slide 4
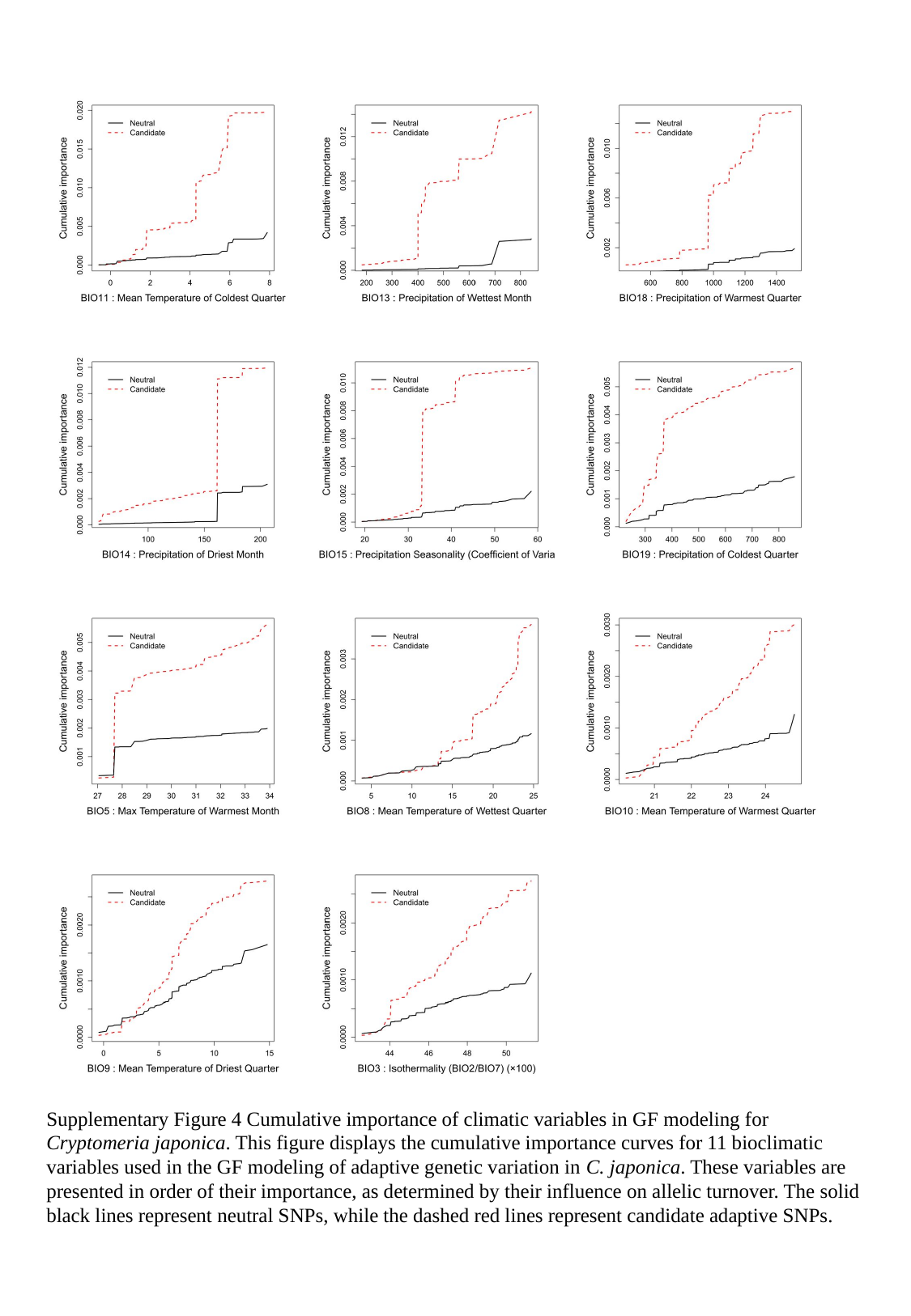

Supplementary Figure 4 Cumulative importance of climatic variables in GF modeling for Cryptomeria japonica. This figure displays the cumulative importance curves for 11 bioclimatic variables used in the GF modeling of adaptive genetic variation in C. japonica. These variables are presented in order of their importance, as determined by their influence on allelic turnover. The solid black lines represent neutral SNPs, while the dashed red lines represent candidate adaptive SNPs.

## Slide 5
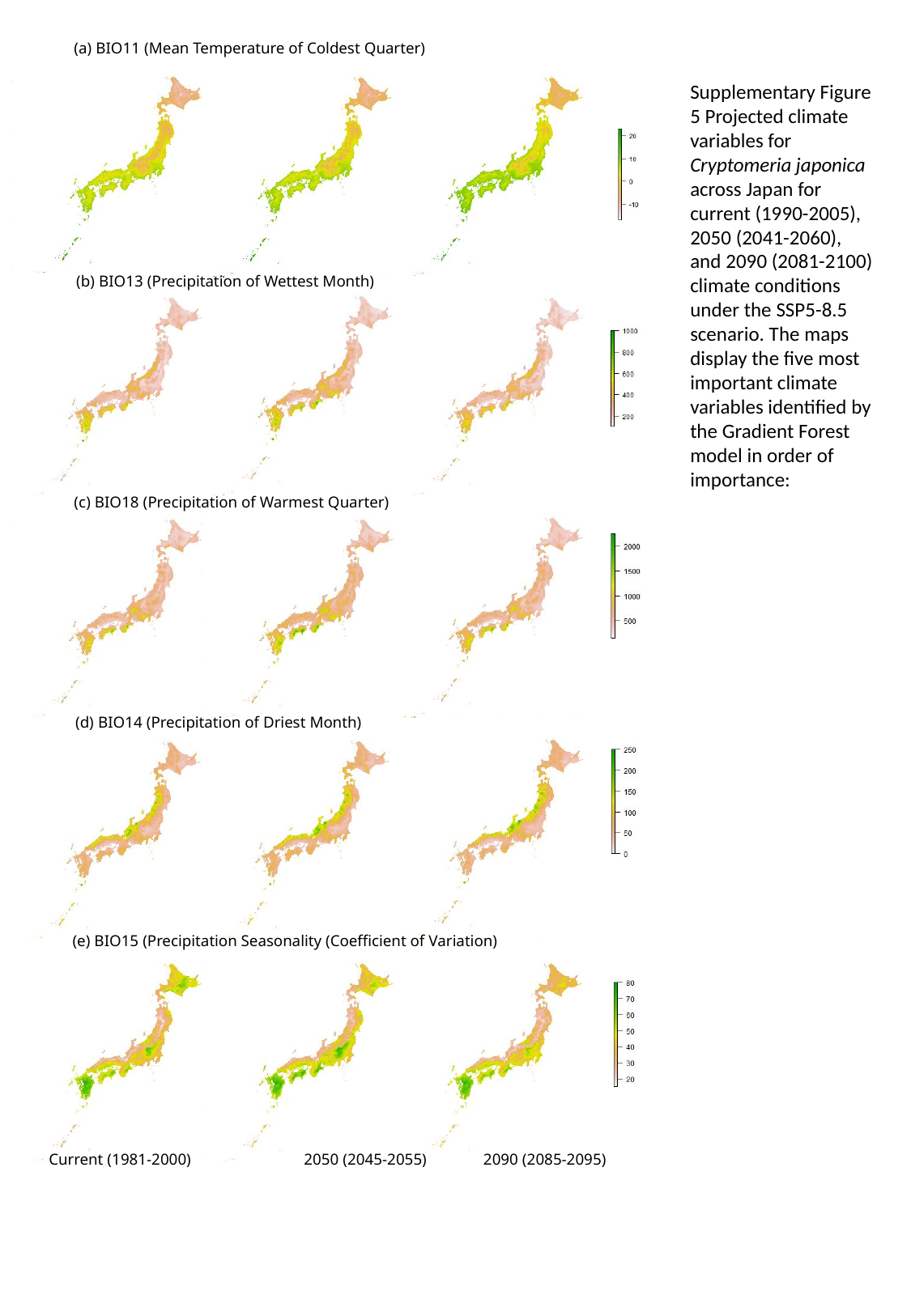

(a) BIO11 (Mean Temperature of Coldest Quarter)
Supplementary Figure 5 Projected climate variables for Cryptomeria japonica across Japan for current (1990-2005), 2050 (2041-2060), and 2090 (2081-2100) climate conditions under the SSP5-8.5 scenario. The maps display the five most important climate variables identified by the Gradient Forest model in order of importance:
(b) BIO13 (Precipitation of Wettest Month)
(c) BIO18 (Precipitation of Warmest Quarter)
(d) BIO14 (Precipitation of Driest Month)
(e) BIO15 (Precipitation Seasonality (Coefficient of Variation)
Current (1981-2000)	 2050 (2045-2055) 2090 (2085-2095)
